# Sono-optogenetics facilitated by a circulation-delivered rechargeable light source for minimally invasive optogenetics

**DOI:** 10.1073/pnas.1914387116

**Published:** 2019-12-06

**Authors:** Xiang Wu, Xingjun Zhu, Paul Chong, Junlang Liu, Louis N. Andre, Kyrstyn S. Ong, Kenneth Brinson, Ali I. Mahdi, Jiachen Li, Lief E. Fenno, Huiliang Wang, Guosong Hong

**Affiliations:** ^a^Department of Materials Science and Engineering, Stanford University, Stanford, CA 94305;; ^b^Wu Tsai Neurosciences Institute, Stanford University, Stanford, CA 94305;; ^c^Department of Chemistry, Stanford University, Stanford, CA 94305;; ^d^Department of Bioengineering, Stanford University, Stanford, CA 94305;; ^e^Department of Psychiatry, Stanford University, Stanford, CA 94305

**Keywords:** optogenetics, ultrasound, minimally invasive neuromodulation, circulatory system, mechanoluminescence

## Abstract

Optogenetics, which uses visible light to control the cells genetically modified with light-gated ion channels, is a powerful tool for precise deconstruction of neural circuitry with neuron-subtype specificity. However, due to limited tissue penetration of visible light, invasive craniotomy and intracranial implantation of tethered optical fibers are usually required for in vivo optogenetic modulation. Here we report mechanoluminescent nanoparticles that can act as local light sources in the brain when triggered by brain-penetrant focused ultrasound (FUS) through intact scalp and skull. Mechanoluminescent nanoparticles can be delivered into the blood circulation via i.v. injection, recharged by 400-nm photoexcitation light in superficial blood vessels during circulation, and turned on by FUS to emit 470-nm light repetitively in the intact brain for optogenetic stimulation. Unlike the conventional “outside-in” approaches of optogenetics with fiber implantation, our method provides an “inside-out” approach to deliver nanoscopic light emitters via the intrinsic circulatory system and switch them on and off at any time and location of interest in the brain without extravasation through a minimally invasive ultrasound interface.

## Significance

Since the invention of optogenetics, using light to control the activity of individual neurons and dissect the complex neural circuits has been a powerful tool for neuroscience owing to the high temporal precision and neuron-type specificity. However, one of the major challenges of optogenetics is the invasive delivery of light sources such as fiber optics inside the brain of live animals due to limited tissue penetration of photons. Here, we report a method termed “sono-optogenetics,” which provides minimally invasive optogenetic neuromodulation in the brain without any scalp incision, craniotomy, or brain implant. Sono-optogenetics delivers nanoscopic light sources via the endogenous blood circulation and provides millisecond-timescale switching of light emission for optogenetic neuromodulation via brain-penetrant focused ultrasound.

Optogenetics is revolutionizing neuroscience research by offering temporally precise and neuron-type-specific dissection of complex neural circuits and brain functions (1–4). Since the first demonstration of optogenetic control of neural activity using channelrhodopsins (5), many efforts have been made to advance both the opsin tools (6–8) and targeting strategies (9, 10). However, one of the existing challenges for in vivo applications of optogenetics is the invasive delivery of light sources in the brain of live animals, which usually involves partial removal of scalp and skull, followed by intracranial implantation of obtrusive optical fibers and light-emitting diodes (LEDs) in the brain tissue (11). Perturbation to the endogenous neural and glial activity has been reported as a consequence of chronic gliosis at the interface and permanent damage of neural tissue due to the invasive craniotomy and implantation procedures (12–15).

The challenge related to the invasive delivery of light source is a direct result of limited tissue penetration of photons for optogenetic stimulation in the brain (16). The conventional optogenetic toolbox comprises opsins with activation spectra in the range of 430 to 610 nm, which has limited tissue penetration due to scattering and absorption of photons by the brain tissue (17). To address this challenge, opsins with red-shifted activation spectra and 2-photon stimulation have enabled optogenetic neural modulation in the intact brain of live animals without any implant (7, 18–20). Another strategy involves the intracranial injection of upconversion nanoparticles, which absorb brain-penetrant near-infrared (NIR) light and emit visible light, into the mouse brain for deep brain optogenetic stimulation by delivering NIR irradiation from an optical fiber placed outside the skull (21, 22). Despite these advances, these methods either require partial removal of scalp and skull to afford deeper penetration (7, 19) or involve intracranial delivery of photoluminescent agents into deep brain tissue (21), while it still remains challenging to achieve in vivo optogenetic stimulation via a much less invasive brain interface without any surgery.

To address these challenges of optogenetics, we replace direct light illumination with focused ultrasound (FUS), the latter of which affords much deeper penetration in biological tissues including the brain (23–28), and replace intracranial delivery of the light source with i.v. delivery, the latter of which is much less invasive and more accessible than the former. Specifically, to address the challenge related to brain penetration, we have developed a form of light source based on mechanoluminescent nanoparticles, which produce strong 470-nm light emission for optogenetic neural stimulation of channelrhodopsin-2 (ChR2) upon excitation of FUS. Nanoparticles have recently been demonstrated to enable novel neural modulation interfaces, owing to their unique capability of converting light and magnetic field into electricity and heat for neural stimulation (29–32). Mechanoluminescent materials, which convert sound into light, are realized in this study by doping ZnS nanoparticles, which are intrinsically photoluminescent with 400-nm photoexcitation, with Ag^+^ and Co^2+^ dopant ions that store the photoexcitation energy until being triggered by FUS. Furthermore, we rationalize that the delivery of light sources, which need to be placed in close proximity to opsin-expressing neurons in the brain, can be changed from the conventional “outside-in” approach to an “inside-out” approach, taking advantage of the endogenous blood vasculature in vivo.

**Fig. 1.**
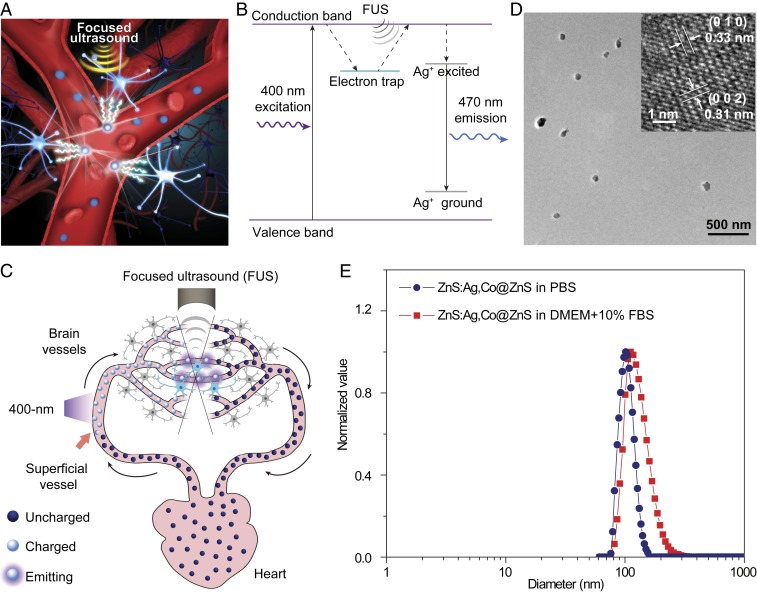
ZnS:Ag,Co@ZnS nanoparticles act as rechargeable light sources in blood circulation for sono-optogenetics. (*A*) Schematic showing sono-optogenetic neural modulation via ultrasound-triggered light emission from ZnS:Ag,Co@ZnS nanoparticles circulating in the blood circulation. (*B*) Mechanism of ultrasound-triggered light emission from ZnS:Ag,Co@ZnS nanoparticles. In this drawing, the electron trap is created in the host material of ZnS by Co^2+^ dopant ions, causing the photoexcited electrons to be trapped after absorption of 400 nm excitation light. FUS allows the trapped electrons to transfer energy into the luminescent centers created by Ag^+^ dopant ions, resulting in 470-nm light emission. (*C*) Schematic showing blood circulation of ZnS:Ag,Co@ZnS nanoparticles, transporting the 400-nm photoexcitation energy at superficial vessels into 470-nm emission in deep-brain regions for optogenetic stimulation. (*D*) Representative TEM image and high-resolution TEM image (*Inset*) of ZnS:Ag,Co@ZnS nanoparticles. (*E*) Distribution of the hydrodynamic diameters of ZnS:Ag,Co@ZnS nanoparticles in PBS (blue) and DMEM supplemented with 10% FBS (red), both revealed by DLS measurements.

The blood vasculature has the following advantages that can be leveraged for minimally invasive delivery of the light source. First, the entire brain is subserved by an interconnected network of cerebral vasculature, which is part of the systemic circulation and ranges from the larger cerebral arteries and venous sinuses to the numerous cerebral capillary vessels. Therefore, delivery of the light source through the blood vasculature enables access to any part of the brain without depth limitation. Second, blood passes through superficial vasculature as it circulates in the body, providing accessible locations shallow enough for 400-nm light to penetrate into these superficial vessels and recharge the circulating light source. Third, the constant pumping of blood into the cerebral vasculature by the heart provides a continuous supply of “fresh” light source into the brain for repetitive optogenetic stimulation. Therefore, the combination of FUS excitation and i.v. delivery of mechanoluminescent nanoparticles offers an advantageous approach of “sono-optogenetics” (Fig. 1*A*), affording fiber-free optogenetics through intact scalp and skull in live animals.

## Results and Discussion

We synthesized mechanoluminescent nanoparticles comprising an Ag/Co-codoped ZnS core and an undoped ZnS shell via a 2-step hydrothermal process (*Materials and Methods*). ZnS is a type II-VI semiconductor with a bandgap energy of 3.7 eV, leading to efficient absorption of ultraviolet light and excitation of electrons to the conduction band (Fig. 1 *B*, *Left* and *SI Appendix*, Figs. S1*A* and S2). Co^2+^ dopant ions in the ZnS matrix create defect states with a trap depth of 0.5 eV below the conduction band, which trap the excited electrons and act as “energy relays” that store the photoexcitation energy without emission (*SI Appendix*, Fig. S1*B*). When FUS is applied, the mechanical stress leads to charge separation in the piezoelectric ZnS matrix, effectively tilting the conduction band and making it easier for the trapped electrons to get “detrapped” and return to the conduction band (Fig. 1 *B*, *Middle* and *SI Appendix*, Fig. S1*C*). After the “detrapping” process, Ag^+^ dopant ions receive the energy transferred from the detrapped electrons (*SI Appendix*, Fig. S1*D*) and produce a 470-nm emission characteristic of Ag luminescent centers as previously reported (Fig. 1 *B*, *Right* and *SI Appendix*, Fig. S1*E*) (33–35). The entire process of photoexcitation, defect-induced trapping, FUS-triggered detrapping, energy transfer, and photoemission can be repeated indefinitely in our mechanoluminescent nanoparticles for repetitive optogenetic stimulation.

The mechanoluminescent materials described above provide an energy relay between the 400-nm photoexcitation and FUS-triggered 470-nm emission, which can be exploited for minimally invasive deep-brain optogenetic neural stimulation via the endogenous blood circulatory system in a living subject (Fig. 1*C*). Specifically, sono-optogenetics is realized with several key design features on the systemic level of the entire organism. First, due to the poor tissue penetration of 400-nm photoexcitation, uncharged mechanoluminescent nanoparticles need to be close enough to the surface of the skin to receive photoexcitation and become charged with energy. Taking advantage of the circulatory system that has blood vessels distributed across various depths in the body, we place the 400-nm excitation light source near the facial artery and jugular vein, which are within a depth of ca. 0.8 mm from the surface of skin in mice (36), for charging the circulating mechanoluminescent nanoparticles when they pass through these illuminated blood vessels (Fig. 1 *C*, *Left*). Second, once charged, these nanoparticles store the energy of photoexcitation and can circulate at any depth inside the body, before releasing the stored energy when triggered by the FUS. Taking advantage of the deep-tissue penetration of FUS, we use a FUS transducer with a center frequency of 1.5 MHz to provide minimally invasive, localized ultrasound stimulation of circulating nanoparticles as they flow past the focus of the applied ultrasound and emit 470-nm light for optogenetic neural stimulation as a result of the mechanoluminescence process (Fig. 1 *C*, *Top*). Third, the constant blood circulation of the body provides a continuous supply of charged nanoparticles to the ultrasound focus in the brain for repetitive optogenetic stimulation within the circulation lifetime of the nanoparticles.

Despite the promise of circulation-delivered rechargeable nanoparticles for minimally invasive optogenetic neuromodulation, one of the challenges we faced was the weak intensity of mechanoluminescence from the ZnS:Ag,Co nanoparticles (37), which was ca. one order of magnitude below the threshold needed for efficient optogenetic stimulation of ChR2 (38). To enhance the emission of ZnS:Ag,Co nanoparticles, we coated the ZnS:Ag,Co nanoparticles with an undoped ZnS shell layer to prevent the luminescence quenching effect caused by the solvent or ligand molecules (39, 40). We carried out comprehensive morphological, size, structural, and spectral characterizations of core-shell ZnS:Ag,Co@ZnS nanoparticles, in comparison with the core-only ZnS:Ag,Co nanoparticles and undoped ZnS nanoparticles for the purpose of luminescence enhancement, with several key findings.

First, core-shell ZnS:Ag,Co@ZnS nanoparticles have a spherical-like morphology with an average diameter of 86.6 ± 13.0 nm (mean ± SD; Fig. 1*D*). High-resolution transmission electron microscopy (TEM) imaging (Fig. 1 *D*, *Inset*) and XRD spectroscopy (*SI Appendix*, Fig. S3) revealed the nanoparticles as wurtzite ZnS. It is noteworthy that wurtzite has a high piezoelectric coefficient due to its noncentrosymmetric structure, leading to stronger mechanoluminescence than zinc blende (35). To render these nanoparticles biocompatible, we used surface modification with an amphiphilic coating of 1,2-distearoyl-sn-glycero-3-phosphoethanolamine-*N*-[methoxy(polyethylene glycol)-2000] (DSPE-mPEG) (*Materials and Methods*). As indicated by the dynamic light-scattering (DLS) measurements (Fig. 1*E*), the surface-modified core-shell ZnS nanoparticles showed good monodispersity with an average hydrodynamic diameter of 101.4 nm in PBS and 111.5 nm in cell medium, in good agreement with the TEM imaging result and the radius of gyration of PEG chains (41).

**Fig. 2.**
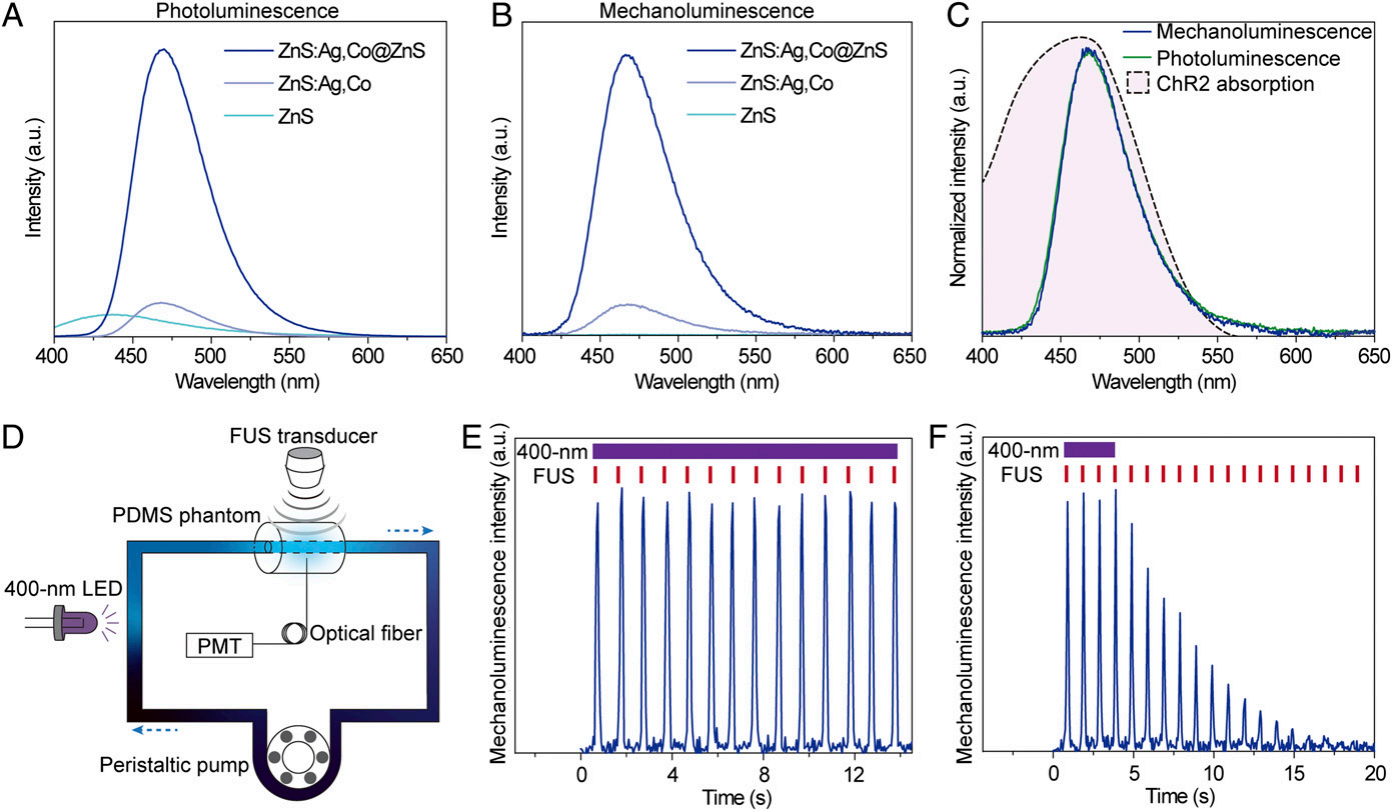
Luminescence properties of ZnS:Ag,Co@ZnS nanoparticles. (*A*) Photoluminescence spectra of undoped ZnS (cyan), ZnS:Ag,Co (gray), and ZnS:Ag,Co@ZnS core-shell nanoparticles (blue) under 365-nm photoexcitation. (*B*) Mechanoluminescence spectra of undoped ZnS (cyan), ZnS:Ag,Co (gray), and ZnS:Ag,Co@ZnS nanoparticles (blue) under FUS excitation. (*C*) Normalized photoluminescence (green) and mechanoluminescence (blue) spectra of ZnS:Ag,Co@ZnS nanoparticles, overlaid with the absorption spectrum of ChR2 (black dashed curve with pink fill). (*D*) Schematic of the artificial circulatory system, where the 400-nm LED provides photoexcitation to charge the circulating ZnS:Ag,Co@ZnS nanoparticles, and the FUS transducer triggers the release of stored energy into 470-nm light emission in the PDMS phantom. (*E*) Intensity of the 470-nm emission from ZnS:Ag,Co@ZnS nanoparticles in the artificial circulatory system under repetitive FUS stimulation (red ticks) and continuous 400-nm recharging light (violet bar). (*F*) Intensity of 470-nm emission from ZnS:Ag,Co@ZnS nanoparticles in the artificial circulatory system under repetitive FUS stimulation (red ticks) and discontinued 400-nm recharging light (violet bar).

Second, we found an 8.6-fold increase of photoluminescence (Fig. 2*A*) and a 9.2-fold increase of mechanoluminescence (Fig. 2*B*) for core-shell ZnS:Ag,Co@ZnS nanoparticles compared to the core-only ZnS:Ag,Co nanoparticles, suggesting effective enhancement of the mechanoluminescence of ZnS:Ag,Co@ZnS nanoparticles that makes them suitable for optogenetic activation of ChR2. The spectra of photoluminescence and mechanoluminescence are found to perfectly overlap with each other after normalization, with the main emission peak located at 470 nm for optimal photoactivation of ChR2, which has a highly overlapping spectral profile of absorbance (Fig. 2*C*). In contrast, the undoped ZnS nanoparticles exhibited blue-shifted photoluminescence with a center wavelength of 430 nm and no detectable mechanoluminescence, suggesting the role of Ag^+^ dopant ions in tuning the emission wavelength to match the absorption profile of ChR2 and the role of Co^2+^ dopant ions to create defect energy states for storing absorbed photoexcitation energy.

The enhanced light emission at 470 nm from core-shell ZnS:Ag,Co@ZnS nanoparticles was bright enough to be visualized under ambient lighting conditions under FUS excitation (*SI Appendix*, Fig. S4). Time-resolved luminescence measurement revealed an 18-fold increase of luminescence intensity upon FUS excitation from the afterglow decay baseline of ZnS:Ag,Co@ZnS nanoparticles (*SI Appendix*, Fig. S5). To test the feasibility of using them as rechargeable light sources in vivo, we made an artificial circulatory system to produce localized photoirradiation in a repeatable and controllable manner (Fig. 2*D* and *SI Appendix*, Fig. S6). The artificial circulatory system comprises several key components to mimic the systemic circulation in a live animal. First, we used a cylindrical piece of polydimethylsiloxane (PDMS) to mimic the biological tissue, with a tunnel inside the PDMS phantom to mimic a deep blood vessel in the brain. Second, both ends of the PDMS-embedded tunnel were connected by a closed loop of Tygon tubing, which was filled with a ZnS:Ag,Co@ZnS nanoparticle suspension to mimic the bloodstream that carries circulation-delivered light source. Third, a 400-nm light source was placed next to the Tygon tubing without illuminating any part of the PDMS phantom (*Materials and Methods*) to recharge the circulating nanoparticles before they enter the PDMS-embedded tunnel and release the stored energy. Fourth, a FUS transducer was placed over the PDMS phantom to excite the circulating nanoparticles inside the tunnel, with a fiber-optic cannula inserted to the opposite end of the PDMS phantom for localized light intensity measurement. Finally, a peristaltic pump was used to mimic the heart that drives the circulation at a constant speed, pumping the charged nanoparticles into the “brain vessel” model for FUS-triggered light emission and the discharged ones back into the circulation for recharging.

We measured the local light emission from circulating nanoparticles in real time when FUS was applied with a repetition rate of 1 Hz (Movie S1) with the following important findings. First, FUS pulses triggered immediate light emission with a short delay of ∼4 ms (*SI Appendix*, Fig. S7). This delay time is shorter than the reported time-to-spike latency time of ChR2 for neural stimulation, the latter of which is above 10 ms (5) and thus does not impose significant delay to the millisecond temporal precision of optogenetic stimulation. Second, repeated FUS excitation demonstrated stable peak intensity of emitted light, in which the variation was found to be within 5% of the peak intensity (Fig. 2*E*), when the 400-nm photoexcitation light source was kept on to continuously charge the nanoparticles in the circulation. This suggests that a steady state of charging by photoexcitation and discharging by FUS was reached for the light source in the circulatory system. Third, when the 400-nm photoexcitation light source was turned off while keeping all other components in the circulatory system unchanged, we found a rapid decrease of the peak intensity of the FUS-triggered mechanoluminescence (Fig. 2*F*), suggesting depletion of the stored energy in the circulating nanoparticles over time. These results suggest that the circulatory system was both necessary and sufficient to deliver charged nanoparticles to the ultrasound focus for repeatable light emission.

**Fig. 3.**
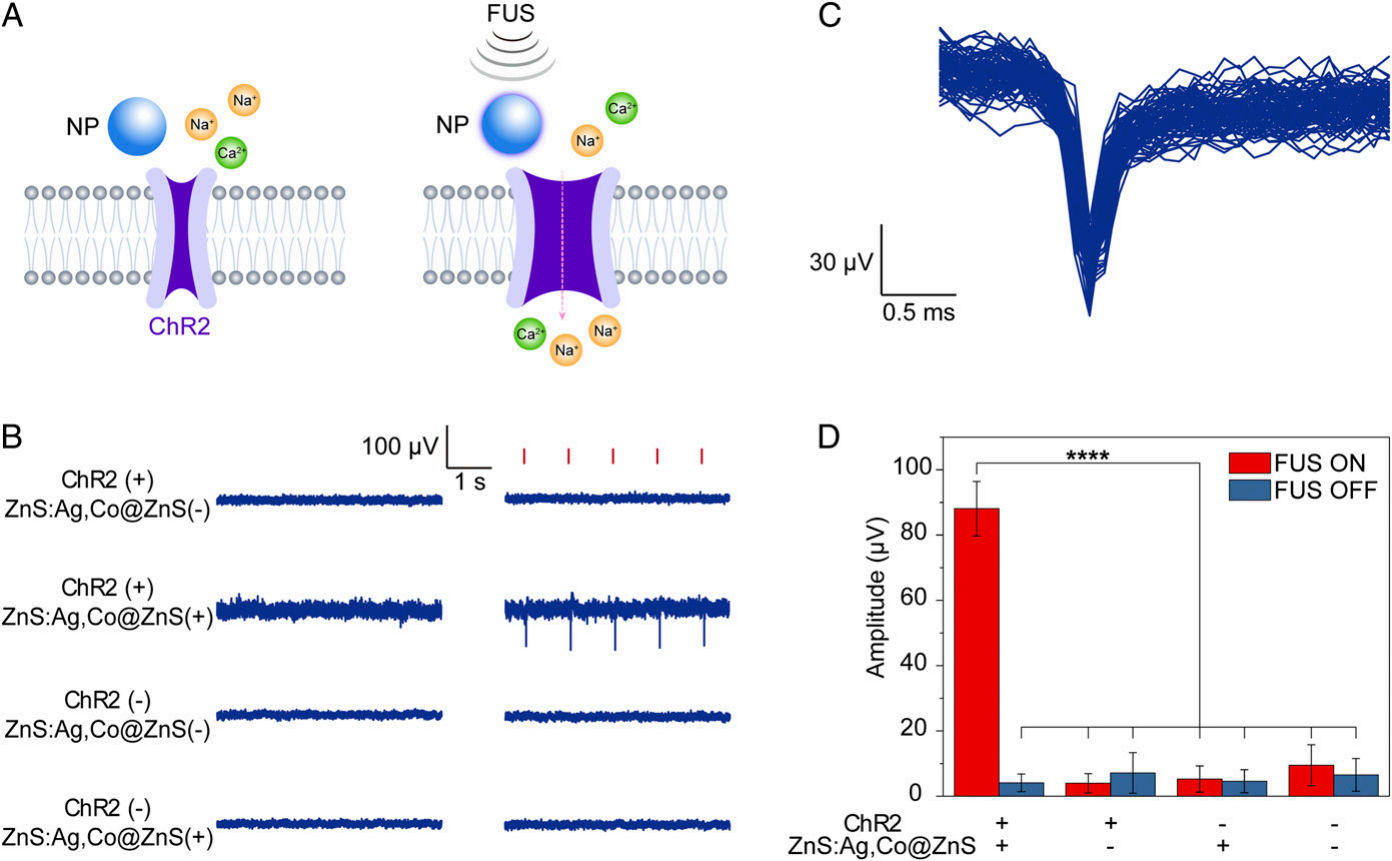
Sono-optogenetic stimulation of spiking HEK cells in vitro. (*A*) Schematics showing ultrasound-triggered opening of ChR2 channels via conversion to 470-nm light emission by ZnS:Ag,Co@ZnS nanoparticles. (*B*) Representative extracellular recording traces of cultured spiking HEK cells under different conditions of ChR2 transfection, ZnS:Ag,Co@ZnS nanoparticle presence, and FUS stimulation. Timed FUS pulses (red ticks) successfully triggered action potentials of spiking HEK cells only when the cells expressed ChR2 and the artificial circulatory system contained ZnS:Ag,Co@ZnS nanoparticles in the circulation. (*C*) Overlaid extracellular single-unit spikes recorded from spiking HEK cells expressing ChR2 and sono-optogenetically stimulated by ZnS:Ag,Co@ZnS nanoparticles and FUS. (*D*) Bar chart summarizing FUS-triggered action potential amplitudes for different groups shown in *C* with FUS on (red bars) and off (blue bars) from *n* = 82 stimuli per group. ****P < 0.0001. The error bars represent ±1 SD.

We then asked whether the artificial circulatory system could be used to evoke action potentials from spiking cells expressing ChR2 under repetitive FUS stimuli. We used NaV 1.3 KIR 2.1 human embryonic kidney (HEK) cells (42) transfected with ChR2 and cultured in a Petri dish for this study. Since the cell medium was stationary and not circulating for replenishment of charged nanoparticles, we assembled an artificial circulatory system to provide continuous 470-nm emission by placing the tubing of the circulatory system between the cell culture and the FUS transducer (*Materials and Methods*). The tubing, which was placed next to the cell culture in this system, acted as the light source for optogenetic stimulation of ChR2 in spiking HEK cells (Fig. 3*A*). We hypothesized that the spiking HEK cells would be triggered by FUS to fire action potentials in synchrony with timed FUS pulses only when the artificial circulatory system was filled with mechanoluminescent nanoparticles and was constantly charged with the 400-nm photoexcitation. Our results of the experiments confirmed the hypothesis with the following key findings. First, extracellular recordings with a microelectrode array (MEA) revealed periodic single-unit action potentials only in the ChR2(+)/nanoparticle(+) group with FUS excitation, in which the spiking HEK cells expressed ChR2 and the artificial circulatory system had circulating mechanoluminescent nanoparticles providing constant 470-nm light emission under FUS (Fig. 3*B*). In contrast, all other groups demonstrated minimal firing activity with FUS excitation, suggesting the lack of direct FUS activation of ChR2. Second, the overlay of triggered action potentials from >80 consecutive FUS stimuli revealed typical waveforms of extracellular single-unit spikes as negative peaks (Fig. 3*C*). The spike amplitude was stably measured under repetitive FUS stimulation, showing a statistically significant difference between the ChR2(+)/nanoparticle(+) group and all other control groups (Fig. 3*D*).

**Fig. 4.**
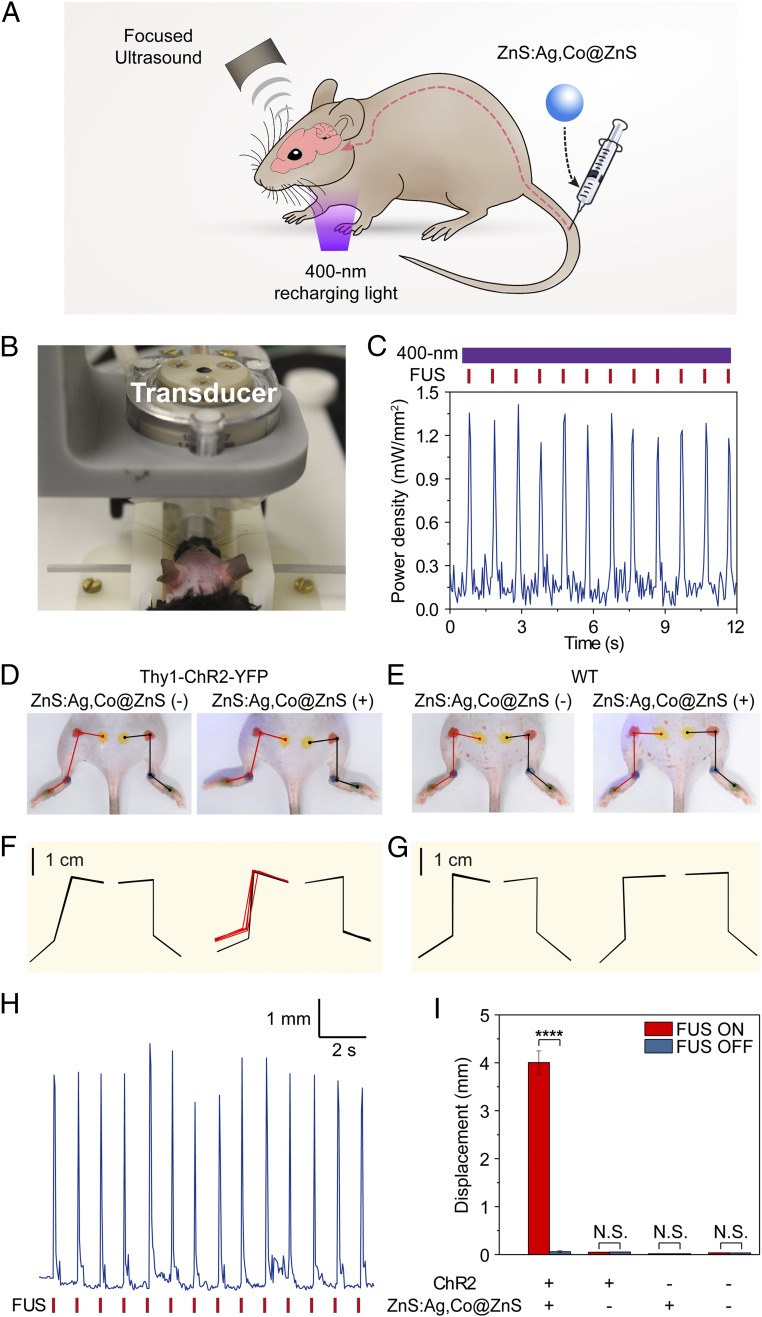
Sono-optogenetic stimulation of motor activity in vivo. (*A*) Schematic of in vivo sono-optogenetic stimulation. (*B*) Photograph of in vivo sono-optogenetic stimulation setup, showing intact scalp and skull of the mouse. (*C*) Measured equivalent power density of 470-nm emission in local brain tissue by circulating ZnS:Ag,Co@ZnS nanoparticles under repetitive FUS stimulation (red ticks) and continuous 400-nm recharging light (violet bar). (*D* and *E*) Photographs of a Thy1-ChR2-YFP mouse (*D*) and a wild-type (WT) mouse (*E*) during sono-optogenetic stimulation through intact scalp and skull, before (*Left*) and after (*Right*) injection of ZnS:Ag,Co@ZnS nanoparticles. Red and black lines indicate the kinematics of left and right hindlimbs, respectively. (*F* and *G*) Hindlimb kinematics of corresponding Thy1-ChR2-YFP mouse (*F*) and WT mouse (*G*) during sono-optogenetic stimulation, with *n* = 4 trials shown for each graph. For each trial, both the starting position and maximum range of motion are shown for each hindlimb, resulting in 8 kinematic diagrams, which are either overlapping or separate depending on the effect of simulation. Contralateral limb activation in the ChR2 mouse by sono-optogenetic stimulation is highlighted in red kinematic diagrams. (*H*) Representative hindlimb displacement over repetitive FUS pulses from a Thy1-ChR2-YFP mouse injected with ZnS:Ag,Co@ZnS nanoparticles. (*I*) Statistics of left hindlimb displacement in different groups of subjects (*n* = 3 per group) in response to FUS excitation. The bar heights indicate the mean, and the error bars indicate SEM. ****P < 0.0001; N.S., not significant.

Having demonstrated rapid, reproducible optogenetic activation of ChR2 in vitro, we asked whether ZnS:Ag,Co@ZnS nanoparticles in the intrinsic circulatory system in live animals allowed for optogenetic stimulation of ChR2-expressing neurons in the brain without craniotomy or any brain implant. We argued that owing to the deep tissue penetration of FUS and the densely distributed cerebral vasculature in the mouse brain, circulation-delivered ZnS:Ag,Co@ZnS nanoparticles could act as localized light sources by providing sufficient 470-nm emission to stimulate ChR2-expressing neurons located in the vicinity of blood vessels without extravasation of the light-emitting nanoparticles, thus allowing for optogenetic stimulation of the brain through intact scalp and skull (Fig. 4*A*). To demonstrate the proof of concept of in vivo sono-optogenetic stimulation, we positioned a Thy1-ChR2-YFP mouse under anesthesia in a stereotaxic frame, exposing the intact scalp in direct contact to a FUS transducer (Fig. 4*B*). We first measured the intensity of 470-nm luminescence emitted from circulating nanoparticles under repetitive FUS excitations and continuous 400-nm recharging light (*Materials and Methods*) to ensure the intensity was sufficient to activate ChR2 in the brain.

Our in vivo luminescence measurements revealed several key findings. First, i.v.-injected ZnS:Ag,Co@ZnS nanoparticles at a concentration of 8 mg/mL in the blood circulation produced 470-nm light emission under FUS excitation, with an equivalent power density of ca. 1.2 mW/mm^2^ (Fig. 4*C*) compared to an intracranially implanted fiber cannula. It has been reported that wild-type ChR2 can be activated with >50% spiking probability under this power density with direct fiber illumination (38). A measured circulation half-life of 127.8 ± 45.3 min suggested that the concentration of circulating ZnS:Ag,Co@ZnS nanoparticles in the bloodstream stayed above 80% of the initial concentration for the first 0.5 h after i.v. injection (*SI Appendix*, Fig. S8). Second, the peak power density of 470-nm mechanoluminescence measured in the brain remained stable over repetitive FUS stimuli, owing to the 400-nm LED positioned near the neck to recharge the circulating nanoparticles in the superficial vessels. Third, the FUS stimuli in our in vivo experiments were measured to have a spatial peak pulsed average intensity $\left(I_{SPPA}\right)$ of 10 W/cm^2^ at the ultrasound focus in the brain tissue (*SI Appendix*, Fig. S9), significantly lower than the safety limit of FUS in mice (43) and the threshold required to provide nonspecific neural stimulation at 1.5 MHz (44). We estimate that with our protocol, the pressure produced by FUS in the brain tissue was able to produce “band tilting” of 0.96 V, sufficient to release the trapped electrons at a depth of 0.5 V to the conduction band for mechanoluminescence emission (35). Fourth, the temperature increase in the local brain tissue with continuous sono-optogenetic stimulation was found to be <0.2 °C over 10 s (*SI Appendix*, Fig. S10), suggesting negligible intracranial heating that would otherwise alter neuronal physiology due to temperature changes (45). These findings suggested the feasibility of using relatively low-power, through-scalp FUS to activate ChR2-expressing neurons and evoke behavioral responses in live animals via circulation-delivered nanoparticles.

Using the parameters determined by the light intensity measurement above, we demonstrated activation of unilateral limb movement by focusing ultrasound to the secondary motor cortex (M2) subserved by blood circulation carrying charged ZnS:Ag,Co@ZnS nanoparticles in the right hemisphere through intact scalp and skull. A video camera was used to track the kinematics of the contralateral and ipsilateral limbs, both of which were marked with dots of different colors at the joints (Movie S2). Our experiments revealed key results that demonstrated unilateral limb activation via sono-optogenetic stimulation. First, the Thy1-ChR2-YFP mouse with ChR2 expressed in the M2 demonstrated obvious hindlimb motion, which was synchronized with the FUS excitation, after injection of ZnS:Ag,Co@ZnS nanoparticles in the blood circulation. The same animal did not exhibit any hindlimb motion before nanoparticle injection under the same stimulation protocol (Fig. 4*D*). Second, the wild-type mouse without ChR2 expression in the brain demonstrated no hindlimb motion, regardless of the presence of mechanoluminescent nanoparticles in the blood circulation (Fig. 4*E*). These results suggested that FUS stimuli alone were unable to evoke limb movement at the specific frequency and power density used in our experiments. It has been reported that ultrasound can stimulate neurons in the brain by modulation of endogenous mechanosensitive ion channels (24, 46); our finding of minimal limb motion with ultrasound alone was likely due to the relatively low power density of FUS at the frequency of 1.5 MHz (44). Third, hindlimb kinematics analysis revealed unilateral motion only in the left hindlimb of the ChR2 mouse in the presence of mechanoluminescent nanoparticles, which was contralateral to the focus of ultrasound in the right hemisphere (Fig. 4 *F* and *G*). The ipsilateral hindlimb showed minimal motion under FUS excitation (*SI Appendix*, Fig. S11). Fourth, quantitative analysis of the contralateral hindlimb kinematics revealed reproducible range of motion over repetitive FUS stimuli (Fig. 4*H*), which is consistent with the stable peak intensity of light emission discussed above (Fig. 4*C*). Finally, our results were successfully reproduced in a group of 3 Thy1-ChR2-YFP mice, with statistically significant difference in comparison with any of the control groups that did not receive injection of ZnS:Ag,Co@ZnS nanoparticles, did not have ChR2 in the brain, or did not have either one (Fig. 4*I*).

Our results of sono-optogenetic stimulation have several fundamental differences from previous reports of nonspecific neural activation with FUS. First, nonspecific ultrasound neuromodulation usually exhibits improved efficacy with low frequencies below 1 MHz, while it has been reported to become increasingly difficult to demonstrate efficacious neuromodulation using ultrasound frequencies above 1 MHz (44, 47). This relationship between ultrasound frequency and neuromodulation efficacy imposes a challenge to spatially confine the FUS in the brain due to the inverse dependence of ultrasound wavelength, which determines the spatial resolution, on frequency. Our method, in contrast, demonstrates efficacious neural stimulation with a center ultrasound frequency of 1.5 MHz, which results in a small in-plane focus of 0.7 mm × 0.7 mm in the *x* and *y* dimensions. Second, a power density of 40 W/cm^2^ was reported for 1.4-MHz ultrasound to achieve a success rate of 50% for muscle contraction in wild-type mice (44). In our experiments, a power density of merely 10 W/cm^2^ at a similar ultrasound frequency of 1.5 MHz was sufficient to activate circulating nanoparticles in the blood and produce enough photon flux to stimulate ChR2 neurons with visible twitches of the hindlimb in a reproducible manner. We reason that the successful sono-optogenetic stimulation under such a low ultrasound power density was owing to the enhancement of mechanoluminescence by coating the ZnS:Ag,Co core with an undoped ZnS shell, as the uncoated ZnS:Ag,Co nanoparticles were unable to elicit any limb motion under the same protocol (*SI Appendix*, Fig. S12). Third, unlike nonspecific neuromodulation with FUS that usually evokes bilateral hindlimb motion (44), our method demonstrates clear contralateral limb activation, owing to the specific expression of ChR2 in Thy1 neurons and spatially confined FUS to afford regional selectivity in the brain. Therefore, sono-optogenetics provides a minimally invasive and cell-type-specific neuromodulation method with high spatial resolution and low power requirement.

Compared to the existing optogenetic methods, sono-optogenetics represents the least invasive technique to implement optogenetic neuromodulation (*SI Appendix*, Table S1). The most common protocol for in vivo optogenetic stimulation involves implantation of a fiber cannula (11, 48) or an LED (49) to the targeted brain region, imposing acute damage to the local neural tissue and chronic gliosis at the fiber interface (50, 51). To mitigate the invasiveness of implantation into the brain, recent advances in implementation of optogenetics take advantage of deeper tissue penetration of longer-wavelength photons by designing red-shifted opsins (7, 18), replacing conventional light sources with upconversion nanoparticles (21), and activating opsins via a 2-photon process (7, 19). Despite these advances, scalp removal and craniotomy are usually required to meet the power requirement and spatial selectivity for optogenetic stimulation in the brain. Our approach represents an example of optogenetic neuromodulation in the brain of live animals without any invasive procedure to the scalp and skull, owing to the deep brain penetration of ultrasound and the unique delivery method of light source via the intrinsic blood vasculature. In our approach, the circulating mechanoluminescent nanoparticles are not required to physically cross the blood–brain barrier for optogenetic stimulation of neurons (Fig. 1*A*), owing to the sufficient penetration depth of 200 µm for 473-nm photons (52) and the pervasive cerebral vasculature penetrating into every region of the brain (53). In comparison to organic mechanophores that emit light during the irreversible break of chemical bonds (28), the rechargeability of ZnS:Ag,Co@ZnS nanoparticles with 400-nm excitation allows for repetitive optogenetic stimulation in the brain after a single i.v. injection, making our method suitable for animal studies that last hours to days. We have also demonstrated the lack of any noticeable tissue damage or pathological lesion in organs of mice injected with ZnS:Ag,Co@ZnS nanoparticles (*SI Appendix*, Fig. S13), suggesting good biocompatibility of our circulation-delivered light sources for sono-optogenetic stimulation.

In summary, we have achieved minimally invasive in vivo optogenetic stimulation in live mouse brain using a sono-optogenetic method. Unlike conventional approaches of light delivery via a brain implant for optogenetic neuromodulation, sono-optogenetics takes advantage of the intrinsic circulatory system to deliver nanosized light sources, the ZnS:Ag,Co@ZnS nanoparticles, and makes use of the brain-penetrant ultrasound to rapidly switch these circulating nanoparticles on and off in specific brain regions. Sono-optogenetics demonstrates efficacious ChR2 activation in vitro and neuromodulation with motor behavioral changes in vivo, the latter of which can be accomplished through intact scalp and skull to minimize any damage to the brain tissue. Engineering of the trap states by varying the dopant ions and dopant concentrations in the nanoparticle matrix could lead to more efficient sono-optogenetic activation of different opsins with less ultrasound power density (35, 54). We envisage that sono-optogenetics provides a unique tool of rapid screening of different target regions in the brain for optogenetic neural modulation, owing to the ease of changing the location of ultrasound focus in the brain by eliminating fiber-optic implantation. Furthermore, sono-optogenetics can be used in other regions of the central and peripheral nervous systems, as well as in other organs such as the heart and lungs, which are usually refractory to fiber implantation due to structural and functional constraints, for precise modulation with optogenetic control of cell activity. In addition, reduction of the footprint and the weight of the ultrasound transducer, as well as the use of ultraflexible neural probes with neural-tissue-like mechanical compliance (55), may enable sono-optogenetic stimulation of deep-brain regions with simultaneous electrophysiology in a behavioral setting. We envision that this approach could also be extended to applications in much deeper brain regions in larger animals owing to the penetration depth of ultrasound reaching several centimeters.

## Materials and Methods

### Synthesis of Mechanoluminescent Nanoparticles.

Chemicals were purchased from Sigma-Aldrich unless otherwise claimed.

#### Synthesis of ZnS:Ag,Co nanoparticles.

The synthesis of ZnS:Ag,Co nanoparticles with afterglow and mechanoluminescence was based on a previous publication with some modifications (34). Zinc acetate (383317; 184 mg, 1 mmol) was weighed and transferred to a 100-mL round-bottom flask followed by dissolution in deionized (DI) water (40 mL) at room temperature by a magnetic stirring hotplate (Cimarec+ Stirring Hotplate; Thermo Fisher Scientific, Inc.). AgNO_3_ (209139; 2.3 mM in DI water, 1.2 mL) and cobalt(II) acetate (399973; 0.8 mM in DI water, 25 µL) were added into the zinc acetate solution and then the solution was stirred at room temperature for 5 min. Complete dissolution of cobalt(II) acetate before transferal to the zinc acetate solution is critical to successful synthesis of ZnS:Ag,Co mechanoluminescent materials, since cobalt(II) acetate is hygroscopic and can hydrolyze into insoluble cobalt(II) hydroxide. Then 3-mercaptopropionic acid (M5801; 0.4 mL) was added into the solution under stirring and the solution became turbid. NaOH (795429; 2 M in DI water) was added into the mixture to adjust the pH to 10.5 and the solution became clear. After that, Na_2_S·9H_2_O (S2006; 0.46 M in DI water, 1 mL) was added into the solution under stirring. The mixture was transferred into a 50-mL Teflon-lined stainless steel autoclave for hydrothermal reaction in an oven (Heratherm OMH60 Lab Oven; Thermo Fisher Scientific, Inc.) at 120 °C for 24 h to yield a white colloid (product **1**) (56). The ZnS:Ag,Co nanoparticles were then purified by addition of 20 mL absolute ethanol followed by centrifugation (8,000 rpm, 8 min). The supernatant was decanted and the nanoparticles were purified further by redispersion into 10 mL DI water by sonication, precipitation by 5 mL ethanol, and then centrifugation (Thermo Scientific Sorvall Legend ×1R Centrifuge; Thermo Fisher Scientific, Inc.) at 8,000 rpm for 10 min. This procedure was repeated twice. The precipitates were dried by lyophilization and transferred into a 5-mL porcelain crucible for calcination in a tube furnace at 800 °C for 3 h under argon atmosphere. The obtained materials were then dispersed in a mixed solvent containing 20 mL absolute ethanol and 10 mL CH_2_Cl_2_ in a 50-mL centrifuge tube and sonicated for 1 h. After that, the dispersion was centrifuged at 1,000 rpm for 10 min. The supernatant was collected and centrifuged at 8,000 rpm for 10 min to collect the precipitates. The precipitates were then redispersed into 30 mL ethanol under sonication and then collected by centrifugation at 8,000 rpm for 10 min. This step was repeated 3 times. The resulting ZnS:Ag,Co nanoparticles were dried by lyophilization to yield a white to off-white powder (yield: 86.5 mg, 88.1%).

#### Synthesis of ZnS:Ag,Co@ZnS nanoparticles with undoped ZnS shell.

Into a 100-mL round-bottom flask with magnetic stir bar was added product **1**, zinc acetate (0.368 g), and Na_2_S·9H_2_O (0.46 M in DI water, 2 mL). After stirring for 5 min at room temperature, the solution was transferred into a 50-mL Teflon-lined stainless steel autoclave for hydrothermal reaction in an oven (Heratherm OMH60 Lab Oven; Thermo Fisher Scientific, Inc.) at 120 °C for 24 h to yield a white colloid of ZnS:Ag,Co@ZnS nanoparticles. Purification and calcination procedures were the same as for ZnS:Ag,Co nanoparticles (yield: 251.5 mg, 85.8%).

#### Synthesis of undoped ZnS nanoparticles without mechanoluminescent properties.

Zinc acetate (383317; 184 mg, 1 mmol) was weighed and transferred to a 100-mL round-bottom flask followed by dissolution in DI water (40 mL) at room temperature by a magnetic stirring hotplate (Cimarec+ Stirring Hotplate; Thermo Fisher Scientific, Inc.). Then 3-mercaptopropionic acid (M5801; 0.4 mL) was added into the solution under stirring and the solution became turbid. NaOH (795429; 2 M in DI water) was added into the mixture to adjust the pH to 10.5 and the solution became clear. After that, Na_2_S·9H_2_O (S2006; 0.46 M in DI water, 1 mL) was added into the solution under stirring. The mixture was transferred into a 50-mL Teflon-lined stainless steel autoclave for hydrothermal reaction in an oven (Heratherm OMH60 Lab Oven; Thermo Fisher Scientific, Inc.) at 120 °C for 24 h to yield a white colloid of ZnS nanoparticles. Purification and calcination procedures were the same as for ZnS:Ag,Co nanoparticles (yield: 84.7 mg, 86.9%).

### Surface Modification of ZnS:Ag,Co@ZnS Nanoparticles.

A total of 100 mg of ZnS:Ag,Co@ZnS nanoparticles and 100 mg of DSPE-mPEG (Avanti Polar Lipids) were added into a 100-mL round-bottom flask followed by the addition of 10 mL dichloromethane (Sigma 270997). The mixture was sonicated for 1 min and then a rotovap was used to remove dichloromethane in the mixture. After that, 20 mL of DI water was added into the dried mixture and DSPE-mPEG–modified nanoparticles were dispersed in water by sonication. The dispersion was then centrifuged at 8,000 rpm for 10 min and the precipitates (DSPE-mPEG–modified nanoparticles) were collected. A total of 20 mL DI water was added into the precipitates and the mixture was sonicated to disperse the nanoparticles.

### TEM Imaging of ZnS:Ag,Co@ZnS Nanoparticles.

Three drops (2.5 µL per drop) of ZnS:Ag,Co@ZnS nanoparticle suspension (200 µg⋅mL^−1^) were deposited on a formvar/carbon film-coated copper grid (Ted Pella, Inc.) and dried in a desiccator for at least 2 h. Afterward, TEM images of ZnS:Ag,Co@ZnS nanoparticles were captured on a Field Electron and Ion Company (FEI) Tecnai Transmission Electron Microscope.

### DLS of ZnS:Ag,Co@ZnS Nanoparticles.

An aliquot of the abovementioned ZnS:Ag,Co@ZnS suspension was diluted with 1× PBS (pH 7.4) and Dulbecco’s Modified Eagle Medium (DMEM) supplemented with 10% FBS to a final concentration of 200 µg⋅mL^−1^. Then, hydrodynamic diameters of diluted ZnS:Ag,Co@ZnS nanoparticles in these 2 solutions were measured by DLS on a Malvern Nano-ZS Particle Sizer (Malvern Panalytical Ltd.).

### UV-Vis-NIR Absorption Spectroscopy of ZnS:Ag,Co@ZnS Nanoparticles.

The UV-Vis-NIR absorption spectrum of the ZnS:Ag,Co@ZnS nanoparticle suspension was measured by a Cary 6000i spectrophotometer (Agilent) with a total path length of 1 mm, background corrected for contribution from water and the cuvette. The measured range was 200 to 800 nm.

### Photoluminescence Spectroscopy of ZnS:Ag,Co@ZnS Nanoparticles.

The photoluminescence spectrum of the ZnS:Ag,Co@ZnS nanoparticle suspension was measured by a Horiba FluoroLog Fluorimeter spectrophotometer (HORIBA Scientific) in a quartz cuvette. The excitation wavelength was 365 nm and the measured range of photoluminescence was 400 to 650 nm.

### Mechanoluminescence Spectroscopy of ZnS:Ag,Co@ZnS Nanoparticles.

ZnS:Ag,Co@ZnS nanoparticles were mixed with PDMS to form a flat, cylindrical phantom (1.6 cm diameter × 0.2 cm thickness) with the nanoparticle concentration of 75 mg⋅mL^−1^. The nanoparticles-containing PDMS sample was clamped and fixed by a custom holder with alligators and placed on top of a FUS transducer coupled with a degassed water bag (Image Guided Therapy) at room temperature, such that the FUS was focused inside and near the upper surface of the phantom. The center frequency of the transducer was 1.5 MHz, and the peak pressure at the focus was 1.86 MPa. A pulse train of 100 ms duration was delivered with a repetition frequency of 1 Hz. During the FUS application, a fiber-coupled spectrometer (OCEAN-HDX-VIS-NIR; Ocean Optics) was used to collect the emitted mechanoluminescence by placing the end of the optical fiber on the upper surface of the phantom opposite the FUS transducer. The spectral range of measurement was 400 to 650 nm, with a wavelength resolution of 0.366 nm and an acquisition time of 4 s.

### Artificial Circulation System to Mimic Blood Circulation.

Tygon tubing (1.5 mm inner diameter [I.D.] and 3.0 mm outer diameter [O.D.]) with a total length of ca. 35 cm was used for making the artificial circulation system to mimic blood circulation in live animals. The tubing was connected to a cylindrical piece of PDMS (2 cm length × 1.2 cm diameter) with a tunnel (diameter = 2 mm) to complete the circulation (*SI Appendix*, Fig. S6). The tubing was filled with a PBS suspension of ZnS:Ag,Co@ZnS nanoparticles at a concentration of 8 mg⋅mL^−1^ to mimic the concentration circulating in the bloodstream. A peristaltic pump (Model 720, Harvard Apparatus) was used to circulate the solution inside the tubing at a rate of 4.75 mL/min. To avoid air bubbles in the circulation system, one end of the tubing was connected to the PDMS tunnel first and the pipeline was filled with 200 µL of ZnS:Ag,Co@ZnS nanoparticle dispersion through one end of the PDMS (liquid inlet), before the other end of the tubing (liquid outlet) was connected to the PDMS tunnel when the pump was running. FUS was applied from the aforementioned transducer at room temperature and delivered to the tunnel inside the PDMS phantom to mimic a deep vessel embedded in the brain tissue. FUS was applied with a repetition frequency of 1 Hz and a duty cycle of 2% for latency time measurement (FUS on, 20 ms; FUS off, 980 ms). All other parameters of FUS are the same as the mechanoluminescence spectroscopy measurement. A 400-nm excitation light was provided from an LED (Mouser Electronics) at a power density of 10.2 mW/mm^2^ to recharge the ZnS:Ag,Co@ZnS nanoparticles while they were circulating in the artificial circulation system. A photomultiplier tube (PMT1001; Thorlabs) was used to collect the emitted mechanoluminescence at 470 nm with a data acquisition rate of 20 Hz, by inserting a fiber-optic cannula with a 400-µm core (Thorlabs), which was connected to the end of the optical fiber, into the piece of PDMS until the end of the cannula was 0.25 mm away from the tunnel.

### Viral Vector Construction.

Viral vectors used in this work include pCMV-hChR2(H134R)-mCherry plasmid which was constructed by Vector Biolabs.

### Cell Culture and Transfection.

NaV 1.3 KIR 2.1 HEK cells with overexpressed voltage-gated sodium channels (NaV) and inwardly rectifying potassium channels (KIR) were purchased from ATCC. Cell culture was maintained in DMEM supplemented with 10% FBS. NaV 1.3 KIR 2.1 HEK cells were transfected with 7.5 µL of lipofectamine 3000 (Invitrogen) with 2,500 ng of total DNA of pCMV-hChR2(H134R)-mCherry plasmids (Vector Biolabs) in Opti-MEM medium (Gibco) and used for in vitro sono-optogenetic stimulation ∼3 to 5 d after transfection.

### In Vitro Sono-Optogenetic Stimulation with the Artificial Circulation System.

NaV 1.3 KIR 2.1 HEK cells transfected with ChR2 or untransfected NaV 1.3 KIR 2.1 HEK cells were plated in a microelectrode array (MEA) (MEA2100 System; Multi Channel Systems MCS GmbH). Two days after plating the cells, the MEA was inspected under an inverted infinity and phase-contrast microscope (Fisher Scientific) to ensure a confluency between 60% and 80% and sufficient coverage of the electrodes with adherent HEK cells. Then the MEA was placed in a headstage (MEA2100-HS; Multi Channel Systems MCS GmbH), which recorded extracellular action potentials from HEK cells during sono-optogenetic stimulation.

An artificial circulation system was assembled with an 8-mm portion of the polyethylene tubing placed between the MEA and the FUS transducer coupled with a degassed water bag. ZnS:Ag,Co@ZnS nanoparticles suspended in PBS solution at a concentration of 8 mg⋅mL^−1^ were loaded into the tubing and allowed to circulate by the peristaltic pump mentioned above. The 400-nm excitation light was confined to the distal end of the circulatory tubing with a power density of 10.2 mW⋅mm^−2^ and covered with black-tape–coated aluminum foil to minimize light leakage to the MEA that would otherwise stimulate ChR2-expressing NaV 1.3 KIR 2.1 HEK cells optically. The distance between the MEA and the FUS transducer was adjusted such that the FUS focus was located near the MEA chamber which the HEK cells adhered to. FUS was applied with a repetition frequency of 1 Hz and a duty cycle of 10% (i.e., FUS on, 100 ms; FUS off, 900 ms), while the MEA headstage measured the extracellular single-unit neuron activity simultaneously. One of the built-in channels in the MEA was routed to connect to the analog output of the function generator that drives the FUS transducer, enabling precise recording of the timestamp when each FUS pulse was turned on. For control experiments in which no ZnS:Ag,Co@ZnS nanoparticles were used as the medium for sono-optogenetic stimulation, PBS solution was used in the artificial circulation system instead (Fig. 3).

### Data Analysis of In Vitro Electrophysiology.

The electrophysiological recording data were analyzed offline. In brief, raw recording data were loaded in a user-written MATLAB program that performs thresholding to extract single-unit spikes. The threshold was set at 50 µV based on estimation of peak amplitudes of measured extracellular action potentials. A 3-ms interval (1 ms before the main peak and 2 ms after the main peak) was used to include the entire waveform of each single-unit spike, and all spikes were overlaid to demonstrate reproducible firing triggered by sono-optogenetic stimulation.

### Vertebrate Animal Subjects.

Adult (20 to 30 g) male C57BL/6J mice (4 wk old; Jackson Laboratory) and Thy1-ChR2-YFP mice (4 wk old; Jackson Laboratory) were the vertebrate animal subjects used in this study. All procedures performed on the mice were approved by Stanford University’s Administrative Panel on Laboratory Animal Care (APLAC). The animal care and use programs at Stanford University meet the requirements of all federal and state regulations governing the humane care and use of laboratory animals, including the USDA Animal Welfare Act, and PHS Policy on Humane Care and Use of Laboratory Animals. The laboratory animal care program at Stanford University is accredited by the Association for the Assessment and Accreditation of Laboratory Animal Care (AAALAC International). Animals were group housed on a 12-h:12-h light:dark cycle in the Stanford University’s Veterinary Service Center (VSC) and fed with food and water ad libitum as appropriate.

### In Vivo Sono-Optogenetic Stimulation with Circulation-Delivered ZnS:Ag,Co@ZnS Nanoparticles.

ZnS:Ag,Co@ZnS nanoparticles were delivered into blood circulation via tail-vein injection. Mice were anesthetized by i.p. injection of a mixture of 16 mg/kg ketamine (KetaVed; Vedco, Inc.) and 0.2 mg/kg dexdomitor (Dexmedesed; Dechra Veterinary Products). The degree of anesthesia was verified via the toe pinch method before the procedure started. To maintain the body temperature and prevent hypothermia of the surgical subject, a homeothermic blanket (Harvard Apparatus) was set to 37 °C and placed underneath the anesthetized mouse (World Precision Instruments, Inc.). Vet ointment (Puralube; Dechra Veterinary Products) was applied on both eyes of the mouse to moisturize the eye surface throughout the experiment. Hair removal lotion (Nair, Church & Dwight) was used for depilation of the mouse head, back, and both hindlimbs. The hair over the mouse head was removed to help form a continuous interface between the scalp and the water bag to reduce reflection of applied ultrasound at the air/skin interface. The hair over the mouse back and hindlimbs was removed to allow for marking the joints of both hindlimbs with different colors (Fig. 4) and tracking the limb trajectories during sono-optogenetic stimulation later. ZnS:Ag,Co@ZnS nanoparticles of which the surfaces were modified with 1,2-distearoyl-sn-glycero-3-phosphoethanolamine-*N*-[methoxy(polyethylene glycol)-2000] (ammonium salt) (Avanti Polar Lipids), dispersed in PBS with a concentration of 80 mg/mL (200 µL), were injected into the mouse through the tail vein by insulin syringes with a 30G needle gauge.

After i.v. injection of ZnS:Ag,Co@ZnS nanoparticles, the mouse was positioned in the built-in stereotaxic frame of the FUS system. The mouse head was fixed by ear bars which are equipped in the animal bed of the FUS system. The FUS transducer (1.5 MHz) integrated with a customized water bag manufactured by Image Guided Therapy was placed on the mouse head with an intact scalp. The stereotaxic coordinates of the secondary motor cortex (M2) are anteroposterior (AP) +1.0 mm, mediolateral (ML) +0.5 mm, dorsoventral (DV) −0.5 mm (57). The height of the water bag was tuned by adjusting the volume of water with a syringe connected to the degassing system. The 400-nm LED (10.2 mW/mm^2^; Mouser Electronics) was positioned near the jugular vessels in the neck of the mouse, with extra caution to ensure no direct illumination of the brain. FUS was applied with a repetition frequency of 1 Hz and a duty cycle of 10% (i.e., FUS on, 100 ms; FUS off, 900 ms), while a video camera was used to capture the motions of both of the mouse’s hindlimbs during sono-optogenetic stimulation. The hindlimb joint locations, which were marked by different-colored dots, were extracted by a user-written MATLAB program for plotting the hindlimb kinematics. Limb displacement was analyzed by computing the maximum displacement of the toe marker (green dot) within each 100-ms pulse of FUS.

### Estimation of Ultrasound Power Density at the Focus in Brain Tissue.

Standard procedures were followed to measure the spatial peak pulsed average intensity $\left(I_{SPPA}\right)$ at the ultrasound focus in the brain tissue (44). Specifically, a hydrophone was used to measure the pressure at the focus of the ultrasound transducer in water. $I_{SPPA}$ in the brain tissue was calculated as

$$I_{SPPA}=\frac{a}{T}\int_{0}^{T}\frac{P^{2}}{\rho v}dt,$$

where *a* is the attenuation of focused ultrasound due to the roof of the mouse skull and is measured as −2 dB (0.63) at 1.5 MHz (44); *T* is the period of a complete pressure waveform applied by the FUS transducer; *P* is the pressure at a particular output amplitude determined by the calibration curve in *SI Appendix*, Fig. S9; ρ is the density of brain tissue (1,040 kg/m^3^); and v is the speed of sound in the brain tissue (1,560 m/s). $I_{SPPA}$ is calculated as 10.0 W/cm^2^ at an output amplitude of 20%, a frequency of 1.5 MHz and a duty cycle of 10%, which are the parameters used in our in vivo experiments.

### Measurement of Luminescence Intensity Triggered by FUS in Brain Tissue.

We measured the intensity of 470-nm luminescence produced by circulating ZnS:Ag,Co@ZnS nanoparticles in cerebral vessels as they were activated by brain-penetrant FUS by inserting a fiber-optic cannula (CFMXD10; Thorlabs) coupled to an optical fiber (M125L01; Thorlabs), which was then connected to a photomultiplier tube (PMT) for power measurement (PMT1001; Thorlabs), into the FUS focus. Unlike conventional light intensity measurement for fiber-coupled optogenetic stimulation, in which the optical fiber outfitted by an LED or a laser module can be directly connected to the power meter for measurement outside the brain before implantation, quantification of light emission power from circulating ZnS:Ag,Co@ZnS nanoparticles could not be performed outside the brain tissue and had to be carried out in situ. However, it was difficult, if not impossible, to accurately measure the light intensity received by neurons in the immediate vicinity of FUS-activated blood vessels, and any measurement by directly placing a power-measuring optical fiber into the brain during FUS application would underestimate the actual power density for the following 2 reasons: First, the intensity of blue light rapidly decays in the brain tissue as a result of endogenous light absorbers and scatterers (16). Second, the optical fiber used for power measurement usually has a small numerical aperture to meet the total internal reflection criterion, leading to inefficient light collection and coupling into the core of the fiber (52).

Therefore, to mitigate these challenges, we inserted 2 fiber-optic cannulas (CFMXD10; Thorlabs), one of which (“the calibration cannula,” M125L01; Thorlabs) was used to deliver 470-nm light from a blue LED (M470F3; Thorlabs) with known power output at the tip of the cannula and the other of which (“the measuring cannula,” M125L01; Thorlabs) was used to collect locally emitted blue photons in the brain tissue, both into the same M2 region of the brain (stereotaxic coordinates: AP +1.0 mm, ML +0.5 mm, DV +0.5 mm). The distance between the ends of the 2 optical fibers was 0.5 mm. In addition, ZnS:Ag,Co@ZnS nanoparticles were delivered into blood circulation at the same dose as described in *In Vivo Sono-Optogenetic Stimulation with Circulation-Delivered ZnS:Ag,Co@ZnS Nanoparticles* above, charged by the 400-nm excitation light source, and activated by FUS to produce local luminescence. When the calibration cannula delivered light and the FUS was off, the output power of the LED was adjusted such that the power measured by the measuring cannula was the same as when FUS was on to trigger emission from ZnS:Ag,Co@ZnS nanoparticles and the calibration cannula delivered no light. Then the power output from the calibration cannula with the matching settings was used to estimate the equivalent power produced by ZnS:Ag,Co@ZnS nanoparticles triggered by FUS.

### Blood Circulation Study.

An amount of 20 μL of blood was collected from the tail vein at various time points (7.5 min, 30 min, 1 h, 2 h, 4 h, 8 h, and 24 h) after injection of 200 μL of 80 mg/mL ZnS:Ag,Co@ZnS nanoparticles into the mouse tail vein (*n* = 3). Each blood sample was diluted 1,000× and added to a quartz cuvette. The photoluminescence spectrum of the diluted blood sample was measured by a Horiba FluoroLog Fluorimeter spectrophotometer (HORIBA Scientific). The peak fluorescence intensity was taken from each spectrum and compared with that of a ZnS:Ag,Co@ZnS nanoparticle solution with known concentrations for determination of the ZnS:Ag,Co@ZnS concentration in the blood samples. A first-order exponential decay was fitted to the data to extract the circulation half-life of i.v.-injected nanoparticles.

### Histological Study.

On day 3 and day 28 after i.v. administration of ZnS:Ag,Co@ZnS nanoparticles, injected mice were euthanized and their major organs (brain, heart, lung, liver, spleen, and kidney) were harvested and fixed in 4% paraformaldehyde. After 48 h of tissue fixation, these organs were embedded in paraffin and sectioned to 10-µm slices. Later, the slices were stained with hematoxylin and eosin (H&E), followed by imaging under an inverted infinity and phase-contrast microscope (Fisher Scientific).

### Data Availability Statement.

The data reported in this paper have been deposited in Zenodo, https://zenodo.org/record/3550221#.XdcT27rwZ1s.

## Supplementary Material

Supplementary File

Supplementary File

Supplementary File
