# Supplementary material for: Sono-optogenetics facilitated by a circulation-delivered rechargeable light source for minimally invasive optogenetics
Source: Proc Natl Acad Sci U S A. 2019 Dec 6;116(52):26332–42. doi: 10.1073/pnas.1914387116 (PMC6936518; doi:10.1073/pnas.1914387116)
Supplement: Supplementary File [file pnas.1914387116.sapp.pdf]

Supplementary Information for:

Sono-optogenetics facilitated by a circulation delivered rechargeable light source for minimally invasive optogenetics

Xiang Wu,[1,3,5] Xingjun Zhu,[1,3,5] Paul Chong,[2,3,5] Junlang Liu,[1,3] Louis N. Andre,[1,3] Kyrstyn S. Ong,[1,3] Kenneth Brinson Jr.,[1,3] Ali I. Mahdi,[1,3] Jiachen Li,[1] Lief E. Fenno,[4] Huiliang Wang[4*] and Guosong Hong[1,3*]

[1] Department of Materials Science andmind Engineering, Stanford University, Stanford, California, 94305, USA

[2] Department of Chemistry, Stanford University, Stanford, California, 94305, USA

[3] Wu Tsai Neurosciences Institute, Stanford University, Stanford, California, 94305, USA

[4] Departments of Bioengineering and Psychiatry, Stanford University, Stanford, California, 94305, USA

[5] These authors contributed equally to this work.

Correspondence should be addressed to guosongh@stanford.edu and whl0903@stanford.edu

This PDF file includes:

Figure S1-S13

Table S1

Legends for Movies S1 to S2

Other supplementary materials for this manuscript include the following:

Movies S1 to S2

Supplementary Figures

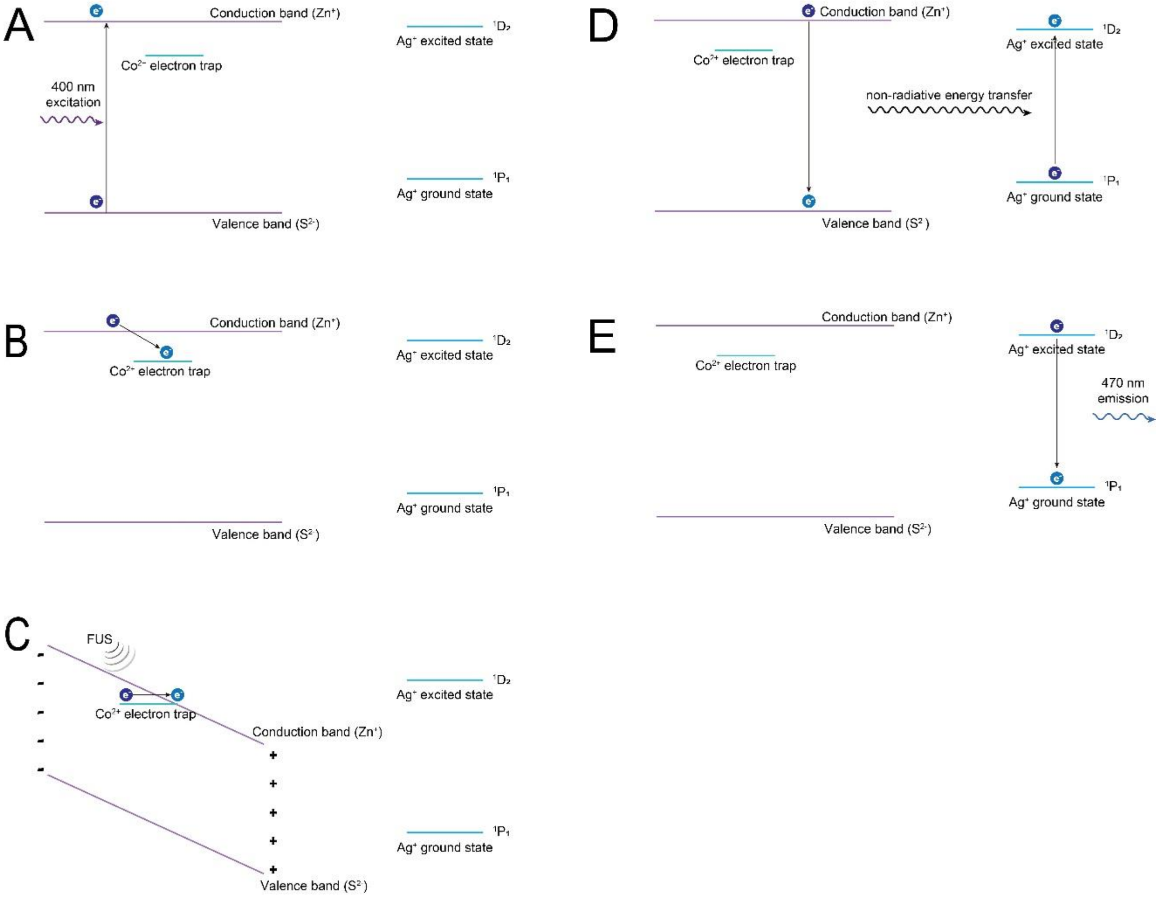

Figure S1. Detailed mechanism of ultrasound-triggered light emission from ZnS:Ag,Co@ZnS nanoparticles. (**A**) ZnS:Ag,Co@ZnS nanoparticles absorb the 400-nm light to excite an electron in the valence band to the conduction band. (**B**) The excited electron is trapped in the defect state created by the Co^{2+} dopant ion. (**C**) Focused ultrasound (FUS) creates mechanical stress and charge separation in the piezoelectric ZnS matrix, effectively tilting the conduction band to equilibrate with the electron trap, thus 'detrapping' the electron back to the conduction band. (**D**) The 'detrapped' electrons transfer energy to the Ag^{+} dopant ions. (**E**) The Ag^{+} dopant ions act as emission centers to release the transferred energy in 470-nm light.

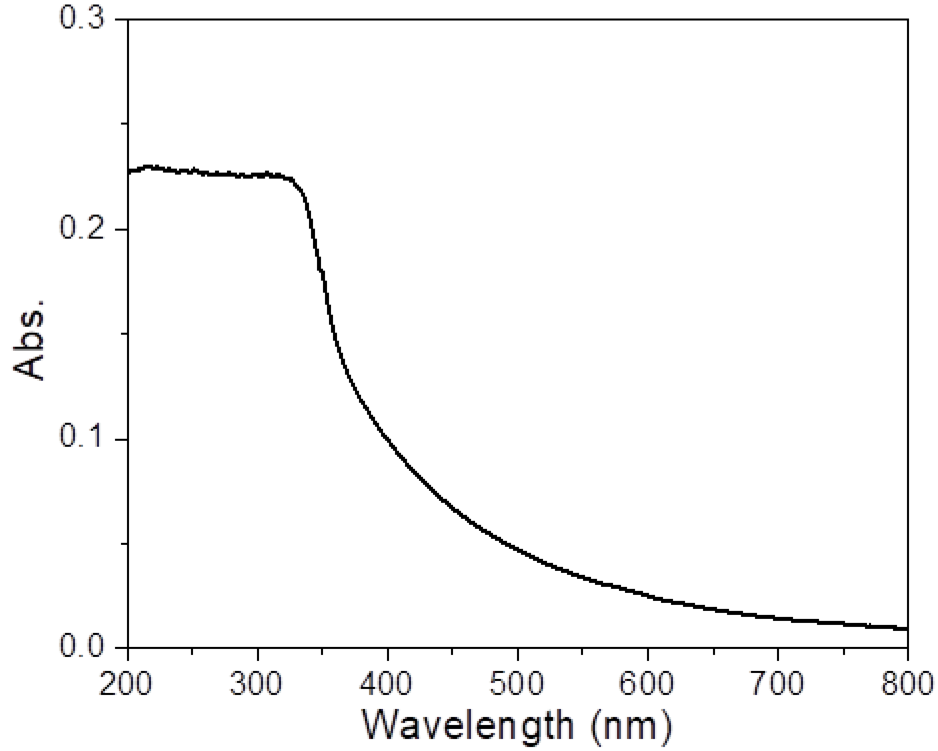

Figure S2. UV-Vis-NIR spectrum of the ZnS:Ag,Co@ZnS nanoparticle suspension.

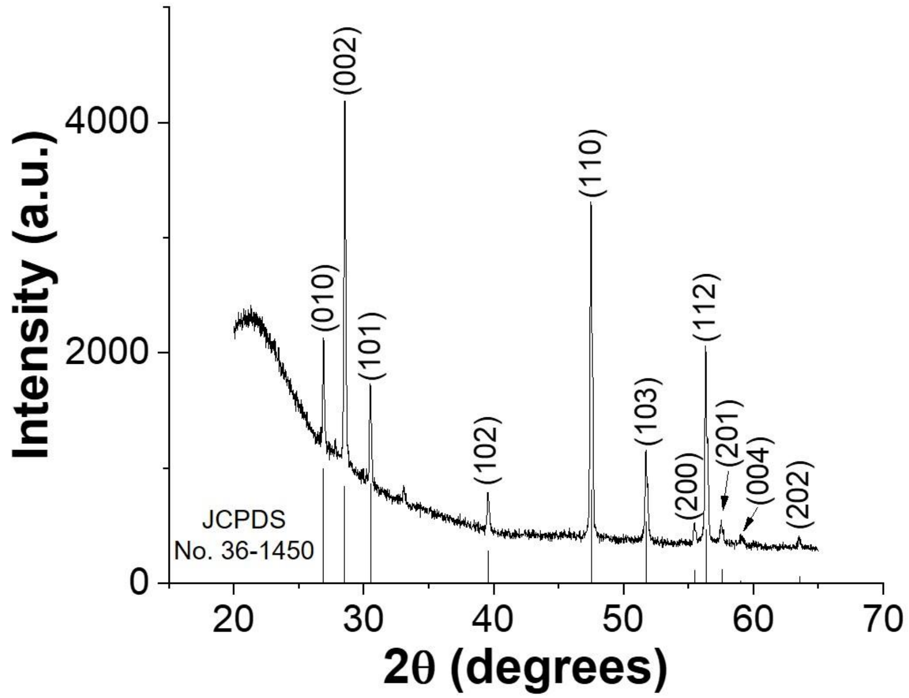

Figure S3. XRD spectrum of the ZnS:Ag,Co@ZnS nanoparticles and the standard peaks reported in the Joint Committee on Powder Diffraction Standards (JCPDS) card no. 36-1450 for wurtzite ZnS.

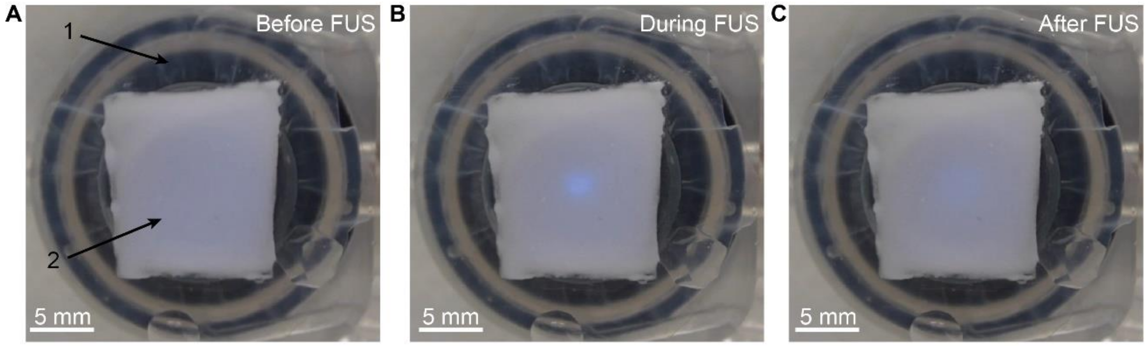

Figure S4. Photographs of a PDMS phantom placed at the focus of the ultrasound transducer, exhibiting minimal emission, visible blue emission and disappearance of the blue emission under ambient lighting before (**a**), during (**b**) and after (**c**) FUS stimulation. Arrow 1 indicates the FUS transducer and arrow 2 indicates the PDMS phantom with embedded ZnS:Ag,Co@ZnS nanoparticles.

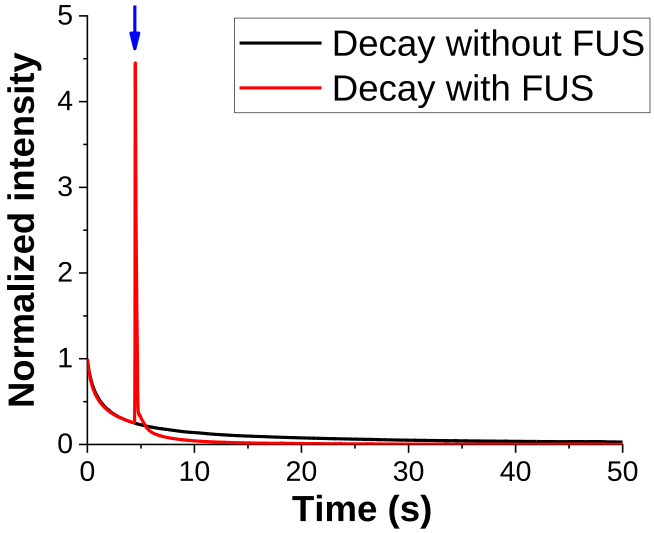

Figure S5. Time-resolved luminescence measurements of the PDMS phantom comprising ZnS:Ag,Co@ZnS nanoparticles, which is shown in **Fig. S4**, with (red) and without (black) FUS excitation after irradiation of 400-nm light (t = 0 s). An exponential decay, which indicates the afterglow of ZnS:Ag,Co@ZnS nanoparticles, is followed by an increase of luminescence intensity by ~18 fold upon FUS excitation at t = 4.5 s (spike in the red curve, indicated by the blue arrow).

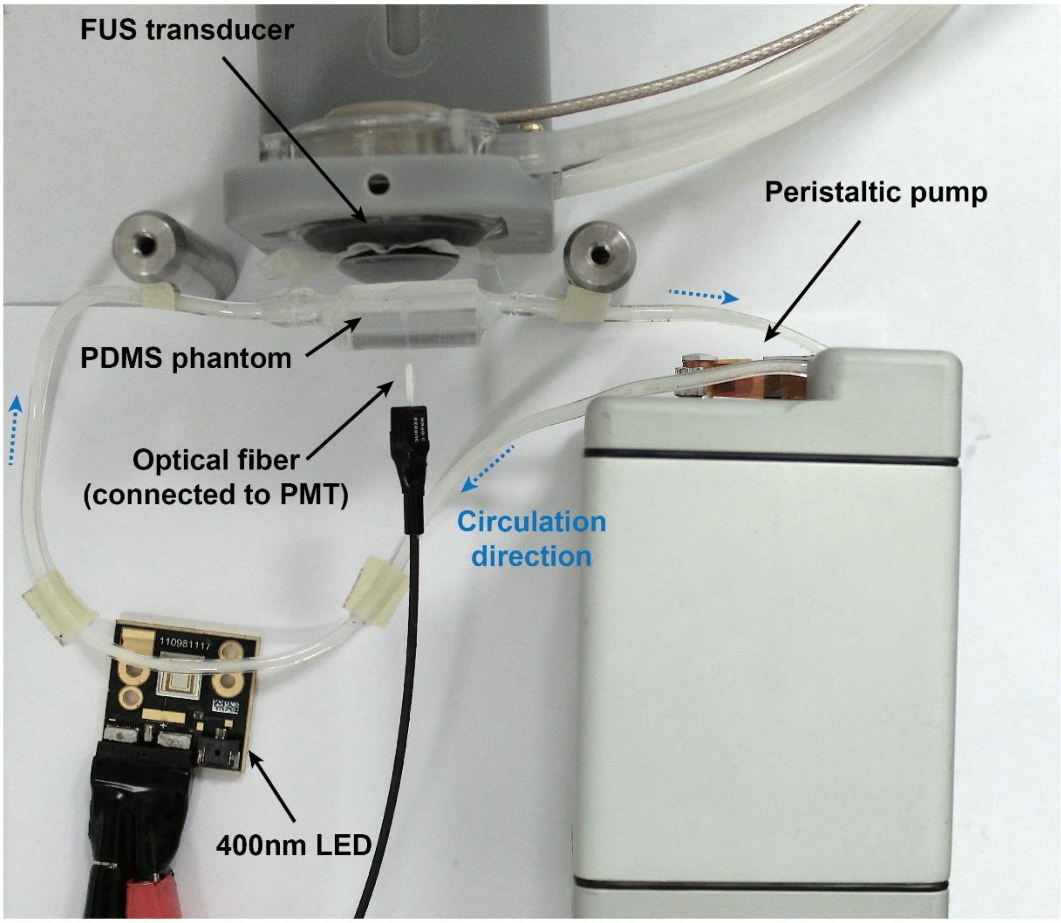

Figure S6. Schematic of the artificial circulatory system for repetitive emission of 470-nm light triggered by FUS and measured by fiber-coupled PMT, as shown in **Fig. 2D-F**.

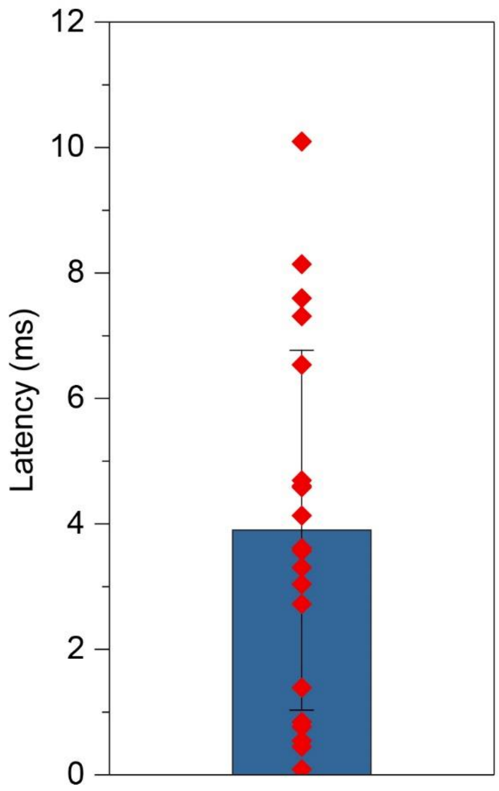

Figure S7. Statistics of latency times between FUS excitation and 470-nm light emission measured from ZnS:Ag,Co@ZnS nanoparticles in an artificial circulatory system. A total of 20 trials are shown in red diamonds, with the height and error bar of the bar chart indicating the mean and standard deviation of measurements, respectively. A sampling rate of 500 Hz along with linear fitting of the rising edge was used for measurement of latency time of mechanoluminescence on the millisecond timescale.

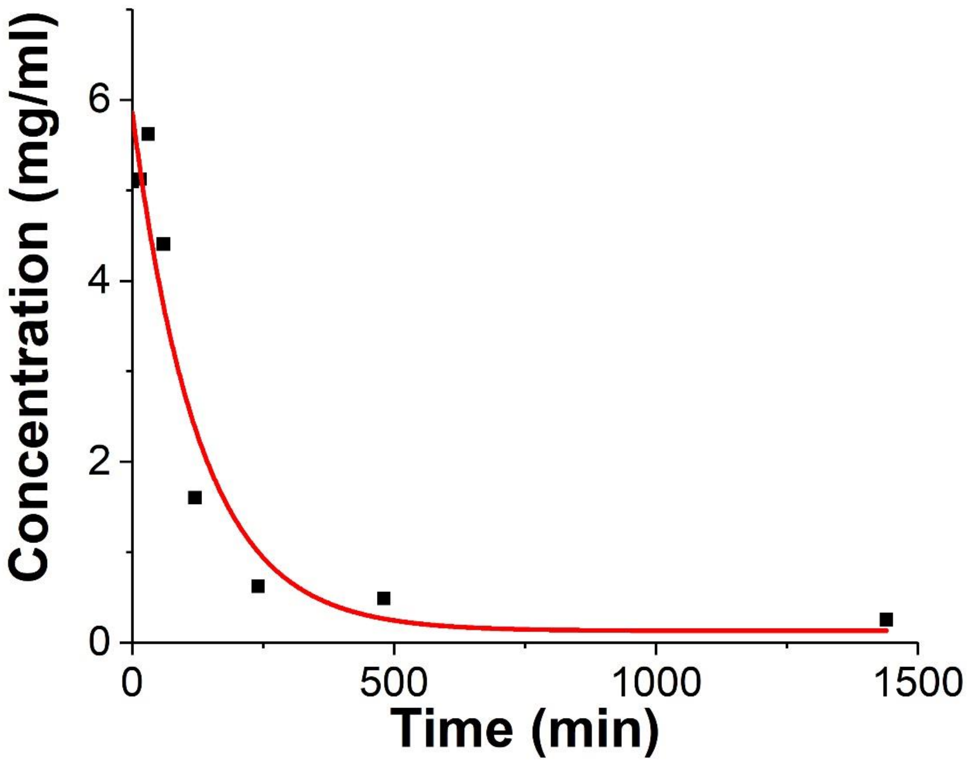

Figure S8. A representative plot of the concentration of the ZnS:Ag,Co@ZnS nanoparticles in the blood versus time after tail-vein injection, as determined by the photoluminescence of the blood samples. A first-order exponential decay fits the data points with a half-life of circulation for the nanoparticles of 127.8 ± 45.3 min.

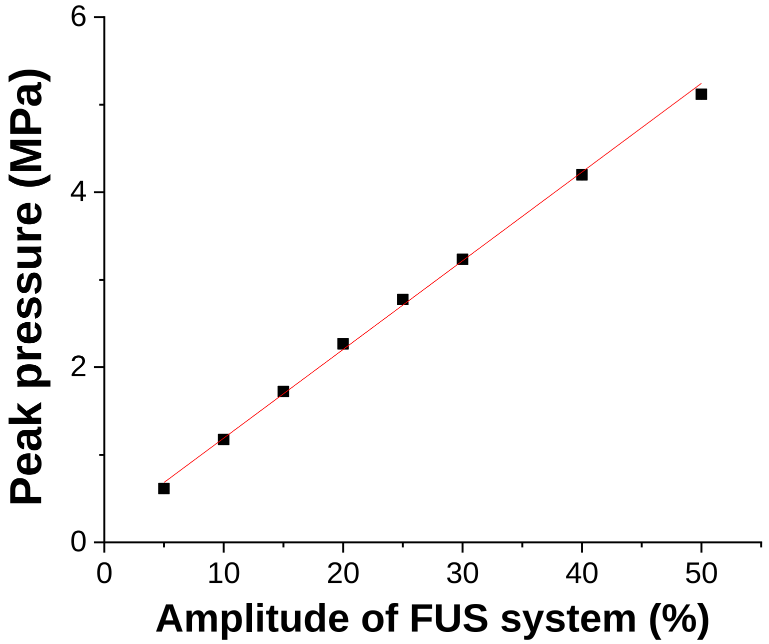

Figure S9. Peak pressure measured by the hydrophone as a function of percent amplitude for the FUS system. Linear fitting results in the empirical formula of $P = 0.101A + 0.178$, where P is pressure in MPa, and A is amplitude in %.

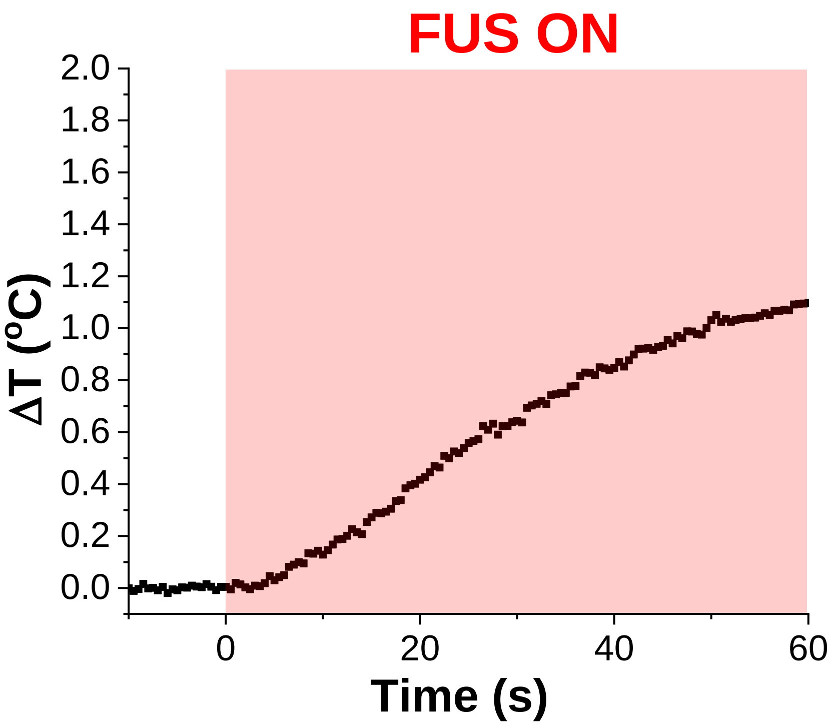

Figure S10. *In vivo* temperature at the ultrasound focus of the mouse brain with the following protocol: FUS frequency: 1.5 MHz; repetition frequency: 1 Hz; duty cycle: 10%; spatial peak pulsed average intensity: 10.0 W/cm^2. Ultrasound was applied after t = 0 s and the duration was indicated with the red shade.

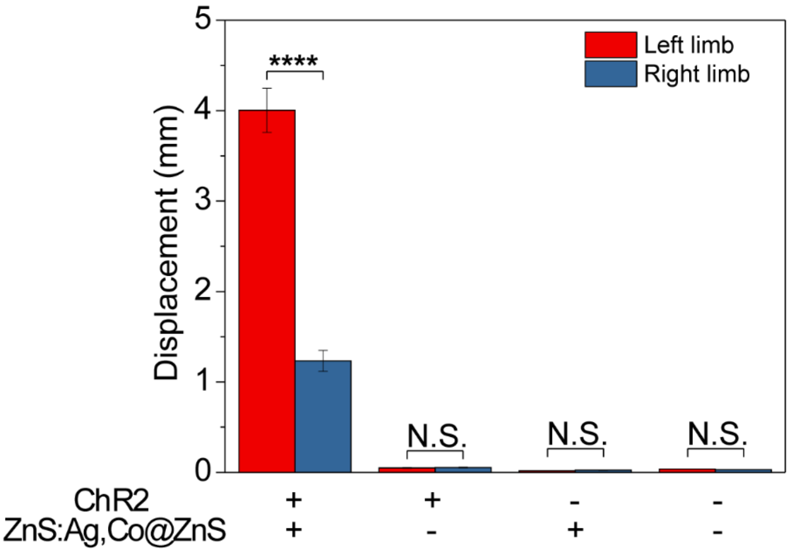

Figure S11. Statistics of left and right hindlimb displacement in different groups of subjects (n = 3 per group) during FUS excitation. The bar heights indicate the mean, and the error bars indicate standard error of the mean (SEM). ****, $p<0.0001$; N.S., not significant.

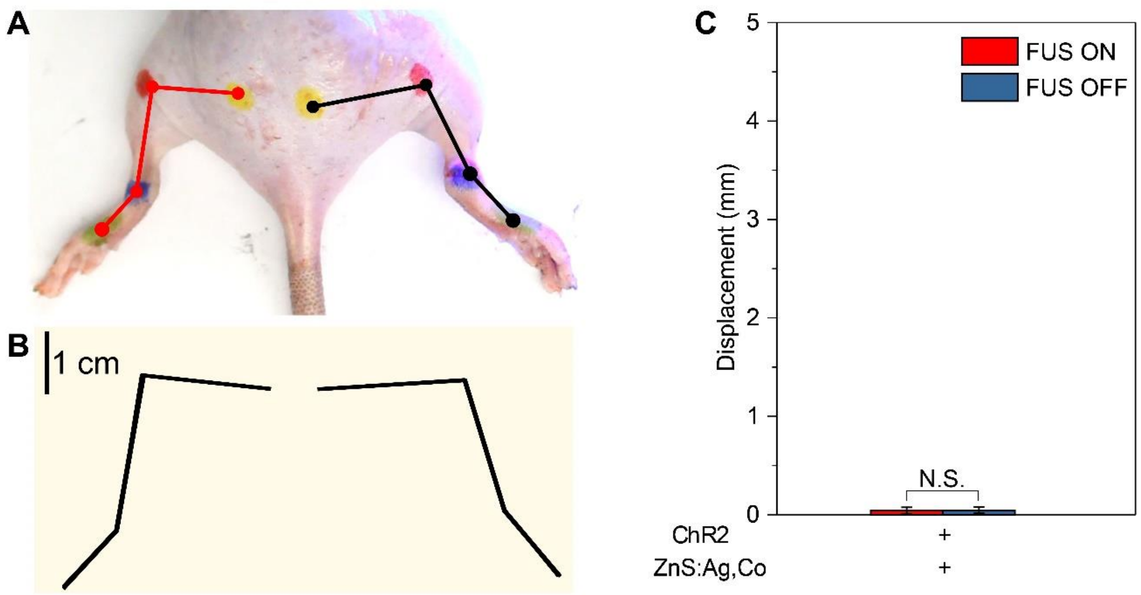

Figure S12. ZnS:Ag,Co nanoparticles without mechanoluminescence enhancement by shell coating do not elicit hindlimb motion under the same sono-optogenetic stimulation protocol (FUS frequency: 1.5 MHz; repetition frequency: 1 Hz; duty cycle: 10%; spatial peak pulsed average intensity: 10.0 W/cm^2). (**A**) Photograph of a Thy1-ChR2-YFP mouse during sono-optogenetic stimulation through intact scalp and skull after injection of ZnS:Ag,Co nanoparticles without the ZnS shell. Red and black lines indicate the kinematics of left and right hindlimbs, respectively. (**B**) Hindlimb kinematics of the mouse shown in (**A**) during sono-optogenetic stimulation over $N = 4$ trials. For each trial, both the starting position and maximum range of motion are shown for each hindlimb, resulting in 8 kinematic diagrams for each limb. (**C**) Statistics of left hindlimb displacement from a group of $n = 3$ Thy1-ChR2-YFP mouse injected with ZnS:Ag,Co nanoparticles with (red) and without (blue) FUS excitation. The bar heights indicate the mean, and the error bars indicate standard deviation (SD). N.S., not significant.

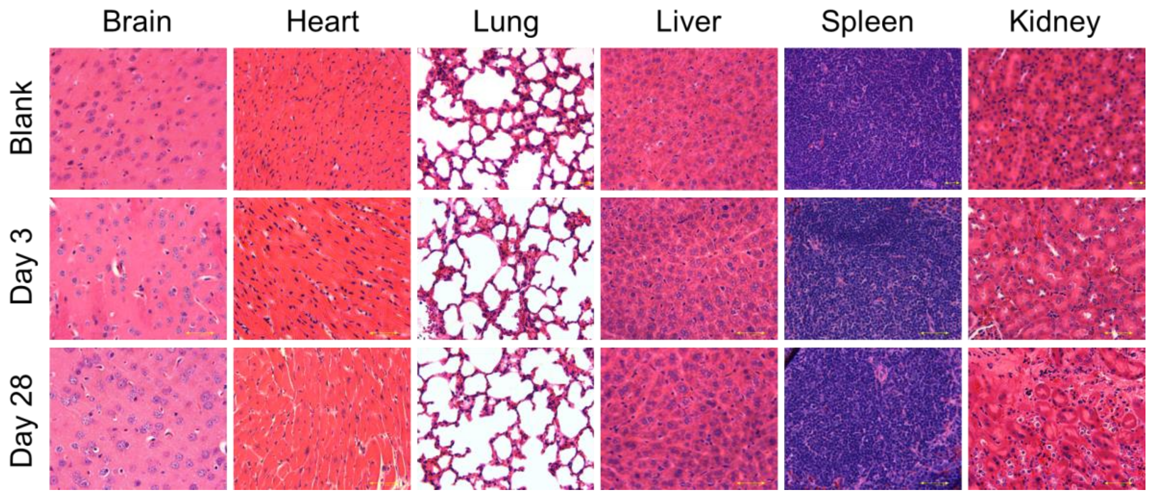

Figure S13. H&E staining of major organs from mice at 3 and 20 days post-intravenous administration of ZnS:Ag,Co@ZnS nanoparticles (middle and bottom rows, respectively) or blank PBS (top row). No noticeable tissue damage or pathological lesion was found in organs from either ZnS:Ag,Co@ZnS nanoparticles or PBS treated groups.

Table S1. Comparison of different methods for *in vivo* optogenetic stimulation.

**Optogenetics stimulation methods**	**Scalp-incised? (+/-)**	**Craniotomy-needed? (+/-)**	**Device-implanted? (+/-)**	**Reference**
Sono-optogenetics	−	−	−	This study
Intracranial fiber	+	+	+	*Nat. Protoc.* **5**, 439–456 (2010).
	+	+	+	*Nat. Neurosci.* **20**, 612-619 (2017).
Intracranial LED	+	+	+	*Neuron* **88**, 1136-1148 (2015).
Two-photon laser	+	+	−	*Nat. Methods.* **9**, 1171–1179 (2012).
	+	+	−	*Science* **365,** eaaw5202 (2019).
Extracranial fiber	+	+	−	*Science* **359**, 679-684 (2018).
Extracranial LED	+	−	−	*Nat. Methods.* **12**, 969-974 (2015).
Red-shifted ChR	−	−	−	*Nat. Neurosci.* **16**, 1499–1508 (2013).

Legends for Supplementary Movies

Movie S1. Focused ultrasound triggers repetitive emission of 470-nm light in an artificial circulatory system. This movie shows the repetitive emission of 470-nm light in the center of the tubing when excited by FUS, which is indicated by the red dot in the upper left corner of the frames. The intensity of 470-nm luminescence is indicated by a colormap ranging from dark blue through blue, cyan, green, yellow to red with increasing intensity. The frame rate is 25 frames per second (fps) and the video is played at real time. Detailed protocol for FUS-triggered mechanoluminescence of artificial circulation system to mimic blood circulation is described in Materials and Methods.

Movie S2. Sono-optogenetic stimulation of mouse motor cortex to evoke contralateral limb movement. This movie shows repetitive FUS excitation of the mouse secondary motor cortex in the right cerebral hemisphere through intact scalp and skull without any craniotomy or brain implant in a Thy1-ChR2-YFP mouse (left column) and a wild-type C57BL/6J mouse (right column) without (top row) and with intravenous injection of ZnS:Ag,Co@ZnS nanoparticles (bottom row). Contralateral limb movement in synchrony with the FUS (indicated by the red dot in the upper left corner) is only found in the Thy1-ChR2-YFP mouse after intravenous injection of the ZnS:Ag,Co@ZnS nanoparticles. The frame rate is 25 frames per second (fps) and the video is played at real time. Detailed protocol for *in vivo* sono-optogenetic stimulation with circulation-delivered ZnS:Ag,Co@ZnS nanoparticles is described in Materials and Methods.
